# Both Transient and Continuous Corticosterone Excess Inhibit Atherosclerotic Plaque Formation in APOE*3-Leiden.CETP Mice

**DOI:** 10.1371/journal.pone.0063882

**Published:** 2013-05-22

**Authors:** Hanna E. Auvinen, Yanan Wang, Hans Princen, Johannes A. Romijn, Louis M. Havekes, Johannes W. A. Smit, Onno C. Meijer, Nienke R. Biermasz, Patrick C. N. Rensen, Alberto M. Pereira

**Affiliations:** 1 Department of Endocrinology and Metabolic Diseases, Leiden University Medical Center, Leiden, The Netherlands; 2 TNO Metabolic Health Research, Gaubius Laboratory, Leiden, The Netherlands; 3 Department of Medicine, Academic Medical Center, Amsterdam, The Netherlands; 4 Einthoven Laboratory for Experimental Vascular Medicine, Leiden University Medical Center, Leiden, The Netherlands; Boston University School of Medicine, United States of America

## Abstract

**Introduction:**

The role of glucocorticoids in atherosclerosis development is not clearly established. Human studies show a clear association between glucocorticoid excess and cardiovascular disease, whereas most animal models indicate an inhibitory effect of glucocorticoids on atherosclerosis development. These animal models, however, neither reflect long-term glucocorticoid overexposure nor display human-like lipoprotein metabolism.

**Aim:**

To investigate the effects of transient and continuous glucocorticoid excess on atherosclerosis development in a mouse model with human-like lipoprotein metabolism upon feeding a Western-type diet.

**Methods:**

Pair-housed female APOE*3-Leiden.CETP (E3L.CETP) mice fed a Western-type containing 0.1% cholesterol for 20 weeks were given corticosterone (50 µg/ml) for either 5 (transient group) or 17 weeks (continuous group), or vehicle (control group) in the drinking water. At the end of the study, atherosclerosis severity, lesion area in the aortic root, the number of monocytes adhering to the endothelial wall and macrophage content of the plaque were measured.

**Results:**

Corticosterone treatment increased body weight and food intake for the duration of the treatment and increased gonadal and subcutaneous white adipose tissue weight in transient group by +35% and +31%, and in the continuous group by +140% and 110%. Strikingly, both transient and continuous corticosterone treatment decreased total atherosclerotic lesion area by −39% without lowering plasma cholesterol levels. In addition, there was a decrease of −56% in macrophage content of the plaque with continuous corticosterone treatment, and a similar trend was present with the transient treatment.

**Conclusion:**

Increased corticosterone exposure in mice with human-like lipoprotein metabolism has beneficial, long-lasting effects on atherosclerosis, but negatively affects body fat distribution by promoting fat accumulation in the long-term. This indicates that the increased atherosclerosis observed in humans in states of glucocorticoid excess may not be related to cortisol *per se*, but might be the result of complex indirect effects of cortisol.

## Introduction

Atherosclerosis develops as a result of a chronic inflammatory response in an injured vessel wall [1], which is preceded by accumulation of leukocytes and fat deposition, leading to plaque formation [2]. The initial mechanisms in atherogenesis, however, are still incompletely understood.

The role of glucocorticoids (GC) in the development of atherosclerosis is not yet clearly established in humans or in animals and is, at least, dependent on individual’s [3], [4] or animal’s [5] exposure to appropriate levels of adrenal steroids. Human data show an association between increased GC secretion and cardiovascular disease even after long-term successful correction of GC excess [6], whereas previous studies in animals, e.g. rabbits [7], [8], [9], [10], [11] and dogs [12] using either natural or synthetic GC, suggest an atheroprotective role of GC. On the other hand, 11β-dehydrogenase type 2 (11βHSD2) deficient mice, in which the activation of the mineralocorticoid receptor (MR) by GCs cannot be prevented, have an increased atherosclerotic plaque development [13], suggesting that increased activation of the MR promotes atherosclerotic plaque formation. However, a recent study demonstrated that adrenalectomy, which removes endogenous GC, stimulated the formation of initial atherosclerotic lesions in low-density-lipoprotein receptor knockout mice [5].

Only a limited number of studies have evaluated the effect of endogenous GC excess on atherosclerosis development in mice. These studies, that used chronic stress to increase endogenous GC, reported either an increase [14], [15] or no effect [16] on atherosclerosis development in ApoE-deficient mice. However, chronic stress, in addition to increasing GC, induces other complex endocrine and metabolic changes, for instance increased sympathetic outflow [17] that may affect atherosclerosis development. These mouse models therefore do not reflect long-term endogenous GC overexposure, like in Cushing’s syndrome (CS) in humans. Furthermore, ApoE-deficient mice do not reflect human-like lipoprotein metabolism and have a deviant immune status compared to wild-type mice [18].

In the present study, our aim was to investigate the effects of GC excess on atherosclerosis development in the APOE*3-Leiden.CETP (E3L.CETP) mouse, a well-established model for human-like lipoprotein metabolism that is prone to develop atherosclerosis upon feeding a cholesterol-containing Western-type diet [19] and is responsive to the hypolipidemic drugs used in the clinic similar to humans [20]–[23] The latter is in sheer contrast to other mouse models for hyperlipidemia and atherosclerosis including apoE-knockout and LDL receptor-knockout mice. We administered corticosterone (CORT) non-invasively via the drinking water, and based on the clinical observation in CS patients, we investigated both transient and chronic effects of CORT on atherosclerosis development.

## Materials and Methods

### Ethics Statement

This study was carried out in strict accordance with the regulations of Dutch law on animal welfare, and the institutional ethics committee for animal procedures of Leiden University. The protocol was approved by the institutional ethics committee for animal procedures of Leiden University (Permit Number: 10132 and for the pilot experiment: 08221) and all efforts were made to minimize suffering.

### Mice, Housing, Corticosterone Supplementation, and Diets

Human CETP expressing transgenic mice, which express CETP under control of its natural flanking regions, were crossbred in our own animal facility with E3L mice to obtain the heterozygous E3L.CETP mice on a C57Bl/6 background [24]. Female mice (10–16 weeks of age) were pair housed and maintained on a 12 h:12 h light-dark cycle (lights on 7 a.m.) in a climate controlled environment, with *ad libitum* access to food and drinking water. Mice were fed a Western-type diet containing 0.1% cholesterol (Diet T +0.1% cholesterol, Arie Blok Diervoeding, Woerden, the Netherlands) for a period of three weeks after which they were matched for age, plasma cholesterol, triglycerides, phospholipids, age and bodyweight and then randomized to receive CORT (Sigma-Aldrich, Manchester, UK) at a concentration of 50 µg/ml in the drinking water with 0.25% ethanol as vehicle for five weeks (transient group, n = 17), continuously for the entire duration of the experiment (seventeen weeks) (continuous group, n = 21), or vehicle (control group, n = 19). Food and bodyweight were recorded weekly. At the end of the experiment mice were decapitated within 90 seconds from disturbing the cage and trunk blood was collected. Gonadal and subcutaneous fat pads were removed from each mouse by the same person to reduce inter-observer variation. The same area of subcutaneous fat was removed from each mouse excluding the inguinal lymph node. Fat pads and adrenals were weighed using a micro scale, and frozen in liquid nitrogen.

### Pilot Experiment for the Determination of Optimal CORT Dose

Prior to experiment, we performed a dose finding study in male C57Bl/6J mice fed HFD with 12.5 µg/ml (n = 2), 25 µg/ml (n = 4) and 50 µg/ml (n = 2) of CORT, and control receiving 0.25% ethanol (n = 2) as vehicle for four weeks to determine the optimal CORT dose. These dosages were chosen based upon a previous study [25] that documented profound metabolic effects with CORT 100 µg/ml and less pronounced effects with 25 µg/ml. Based upon our dose finding study, we chose 50 µg/ml CORT in the drinking water for our subsequent experiments as this dose led to the largest increases in food intake and body weight as well as in plasma cholesterol. Because male mice do not readily develop atherosclerosis and the known models are in majority female models, we used female mice for the purpose to study the atherosclerosis development. In males and females, this CORT dose was sufficient to increase food intake and to maintain a higher bodyweight throughout the experiment. In addition, circulating circadian CORT levels at week 5 were 5–8 fold increased in the morning and 3–4 fold in the evening (data not shown).

### Sampling of Circadian Corticosterone, Hormone, and Lipid Measurements

Plasma CORT was sampled before CORT administration (baseline), and after CORT administration at week 5 during the first light hour at 07.00 h, at 12.00 h, during the last light hour at 18.00 h, and three hours after the onset of the dark phase at 22.00 h. During the dark phase samples were collected in red light conditions. All CORT samples were obtained within 90 seconds from disturbing the cage, via tail incision, allowing the mouse to move freely on top of the home cage [26]. Trunk blood was used to determine plasma CORT at the end of the experiment after CORT administration at week 17 at 09.00 h and 18.00 h. Total plasma cholesterol, triglycerides, phospholipids, insulin and glucose were sampled after 4 hour-fast at baseline, week 5, 8 and 17 of the intervention. Body weight and food intake were measured weekly.

Plasma CORT levels were determined by radioimmunoassay (MP Biomedicals LCC, Orangeburg, NY). Plasma levels of total cholesterol, triglycerides and non-esterified free fatty acids were measured with enzymatic colorimetric reaction (Roche diagnostics GmbH, Mannheim; and Wako Pure Chemical Industries, respectively), plasma insulin was measured with an ELISA (Crystal Chem Inc., Downers Grove, IL, USA) and plasma glucose with a hexokinase method (Instruchemie, Delfzijl, The Netherlands). Homeostasis model index of insulin (HOMA-IR) was calculated by multiplying fasting insulin concentration (µU/ml) with fasting glucose (mmol/l), and dividing with 22.5 [27].

### Lipoprotein Profiling

Distribution of cholesterol over plasma lipoproteins was determined using fast protein liquid chromatography. Pooled plasma from each group were used and 50 µl of each pool was injected onto a Superpose 6 PC 3.2/30 column (Äkta System, Amersham Pharmacia Biotech, Piscataway, NJ) and eluted at a constant rate of 50 µl/min in PBS, 1 mM EDTA, pH 7.4. Fraction were collected and assayed for cholesterol as described above.

### Gene Expression Analysis in Adipose Tissue

Total RNA was extracted from gonadal fat pads using the Nucleospin RNA II kit (Macherey-Nagel, Duren, Germany) according to manufacturer’s instructions. RNA quality of each sample was examined by the lab-on-a-chip method using Experion Std Sens analysis kit (Biorad, Hercules, CA) and RNA concentration of each sample was determined by Nanodrop technology (Thermo Scientific, Wilmington, USA). Then, total RNA was reverse-transcribed with iScript cDNA synthesis kit (1708891, Bio-Rad), and obtained cDNA was purified with Nucleospin Extract II kit (636973, Macherey-Nagel, Bioké). Real-time qPCR was performed on a CFX96 machine (Bio-Rad), the reaction mixture consisting of SYBR-Green Sensimix (QT615, GC Biotech), cDNA, primers (Biolegio, Nijmegen, The Netherlands), and nuclease-free water in a total reaction volume of 10 µl. mRNA values of each gene were normalized to mRNA levels of ß2-microglobulin (*ß2m*) and hypoxanthine ribosyltransferase (*Hprt*). Primer sequences are listed in Table S1.

### Quantification of Atherosclerosis

After 17 weeks of intervention, mice were killed by decapitation and the hearts were isolated. Hearts were fixed in phosphate-buffered 4% formaldehyde, dehydrated and embedded in paraffin. Cross-sections (5 µm) throughout the aortic root area were cut. 12 sections per mouse, stained with hematoxylin-phloxin-saffron for histological analysis, with 50 µm-intervals were used for atherosclerosis measurements. Lesions were categorized for severity according to the guidelines of the American Heart Association, adapted for mice [28], [29], as follows: type 0 (no lesions), types 1 through 3 (early fatty streak-like lesions containing foam cells), and type 4 to 5 (advanced lesions containing foam cells in the media, presence of fibrosis, cholesterol clefts, mineralization, and/or necrosis). AIA 31240 antiserum (1∶3000, Accurate Chemical and Scientific, Westbury, NY) was used to quantify macrophage content of the plaque as well as monocytes adhering to the endothelium. Lesion area, the macrophage area and the number of monocytes adhering to the endothelium were quantified using Image J software (National Institutes of Health).

### Serum Macrophage Colony-stimulating Factor (M-CSF) and Anti-oxidized Low-density Lipoprotein (ox-LDL) Antibodies Measurements

Macrophage colony-stimulating factor (M-CSF) was measured using a mouse M-CSF Quantikine ELISA kit according to the manufacturer’s instructions (MMC00, R&D Systems Inc, Germany). An EIA/RIA high binding 96-well Costar plate (Corning Inc., Corning, NY, USA) was coated with ox-LDL (7.5 µg/mL) in PBS. IgM and IgG2a antibodies against oxLDL in serum were measured using an ELISA Ig detection kit (Zymed Laboratories, San Francisco, CA, USA) according to the manufacturer’s protocol.

### Statistical Analysis

Data are presented as means ± SEM. Statistical differences were calculated using Anova with Tukey’s post-hoc test, for multiple comparisons, except for plasma CORT measurements transient and continuous groups were compared with the control group individually per time point using an unpaired two tailed T-test, with GraphPad Prism, version 5.01. *P*<0.05 was considered as statistically significant.

## Results

### CORT Treatment Increases Plasma CORT Concentrations and Affects Circadian Rhythm

**Figure 1:**
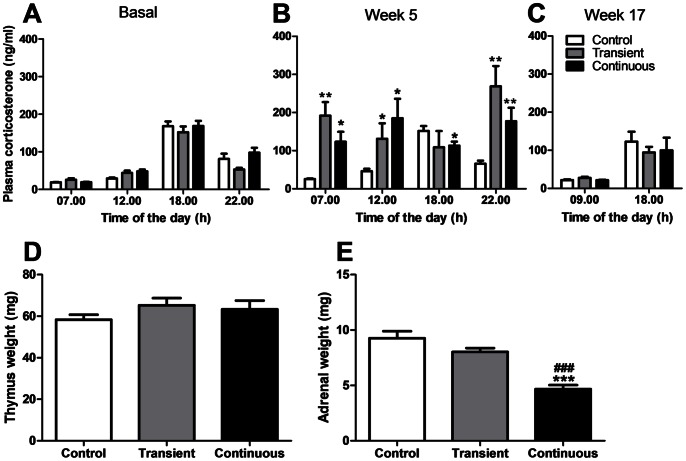
Effect of transient and continuous CORT treatment on circadian plasma CORT levels in female E3L.CETP mice at baseline (A), week 5 (B) and week 17 (C), as well as on thymus weight (D) and adrenal weight (E) at week 17 (Control group: white bars, transient group: grey bars and continuous group: black bars). Data are means ± SEM (n = 17–21), *^, #^ *P*<0.05, **^, ##^ *P*<0.01, ^###^ *P*<0.0001, *versus control group and ^#^transient group.

Chronic administration of high doses of CORT in the drinking water (50 µg/ml) resulted in significant increases in plasma CORT levels at week 5 (Fig. 1B) in both groups, compared to controls (transient group: 07.00 h 8-fold, 12.00 h 3-fold, 18.00 h 1-fold and 22.00 h 4-fold; continuous group: 07.00 h 5-fold, 12.00 h 4-fold, 18.00 h 1-fold and 22.00 h 3-fold). At week 17 (Fig. 1C) there were no differences between groups at 09.00 h and 18.00 h. At the end of the experiment, thymus weight (Fig. 1D) was not different between the three groups but adrenal weight (Fig. 1E) was significantly reduced in the continuously exposed group by −50%, in agreement with adrenal atrophy secondary to long-term exogenous GC exposure.

### CORT Treatment Affects Food Intake and Bodyweight and Induces Long-lasting Changes in Body Composition and Inflammation in Adipose Tissue

**Figure 2:**
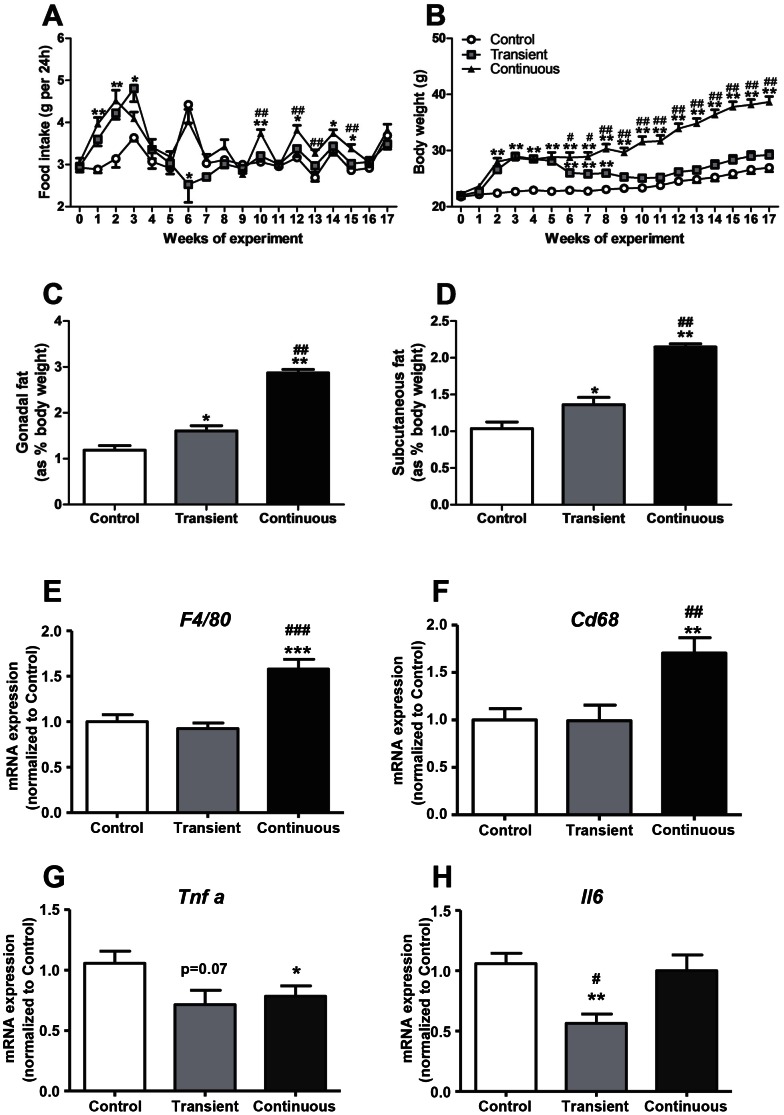
Effect of transient and continuous CORT treatment on food intake (A), body weight (B) (Control group: white circles, transient group: grey squares and continuous group: black triangles), gonadal fat (C) and subcutaneous fat (D) as % of the body weight, mRNA expression of ***F4/80*** **(E),** ***CD68*** **(F),** ***Tnfα*** **(G) and** ***Il-6*** **(H) in the gonadal fat (Control group: white bars, transient group: grey bars and continuous group: black bars).** Data are means±SEM (n = 17–21), Anova with Tukey’s post-hoc test, *^, #^ *P*<0.05, **^, ##^ *P*<0.01, ***^, ###^ *P*<0.001, *versus control group and ^#^versus transient group.

As expected, CORT treatment increased food intake of the transient and the continuous group during the first three weeks of the experiment after which the transient group returned to the level of the controls and food intake with continuous treatment remained elevated (Fig. 2A). This increase was accompanied by an increase in body weight (Fig. 2B) in both groups. After the discontinuation of CORT treatment, body weight decreased to the level of the controls. The continuously exposed group showed a continuous increase in body weight and maintained a higher body weight to the end of the experiment (Fig. 2B) compared to the other two groups. After 17 weeks, gonadal and subcutaneous fat pad weights (Fig. 2C and D), when compared to controls, were significantly increased by +35% and +31%, respectively, in the transient group, and by +140% and +110% in the continuous group. To evaluate whether the increased fat mass resulted in changes of inflammation in the fat pad, the mRNA expression of markers of the macrophage content (i.e. *F4/80* and *Cd68*) and proinflammatory cytokines [Tumor necrosis factor α (*Tnfα*) and Interleukin-6 (*Il-6*)] in the gonadal fat pad were determined. As compared to control group, transient administration of CORT did not affect the expression of *F4/80* (Fig. 2E) and *CD68* (Fig. 2F), but decreased the expression levels of *Tnfa* (Fig. 2G) and *Il-6* (Fig. 2H) in the long-term by −32% and −47%, respectively; while continuous administration of CORT increased the expression of *F4/80* (Fig. 2E) and *CD68* (Fig. 2F) by +58% and +70%, respectively, decreased the expression *of Tnfα* (Fig. 2G) by −26% and did not affect the expression of *Il-6* (Fig. 2H). These data indicate although excess GC exposure increased fat mass, which was accompanied with an increase in macrophage content, expression of proinflammatory cytokines in the adipose tissue was generally reduced.

### CORT Treatment does not Affect Plasma Lipids, Cholesterol Lipoprotein Profile, but Increases Plasma Insulin and HOMA-IR

**Figure 3:**
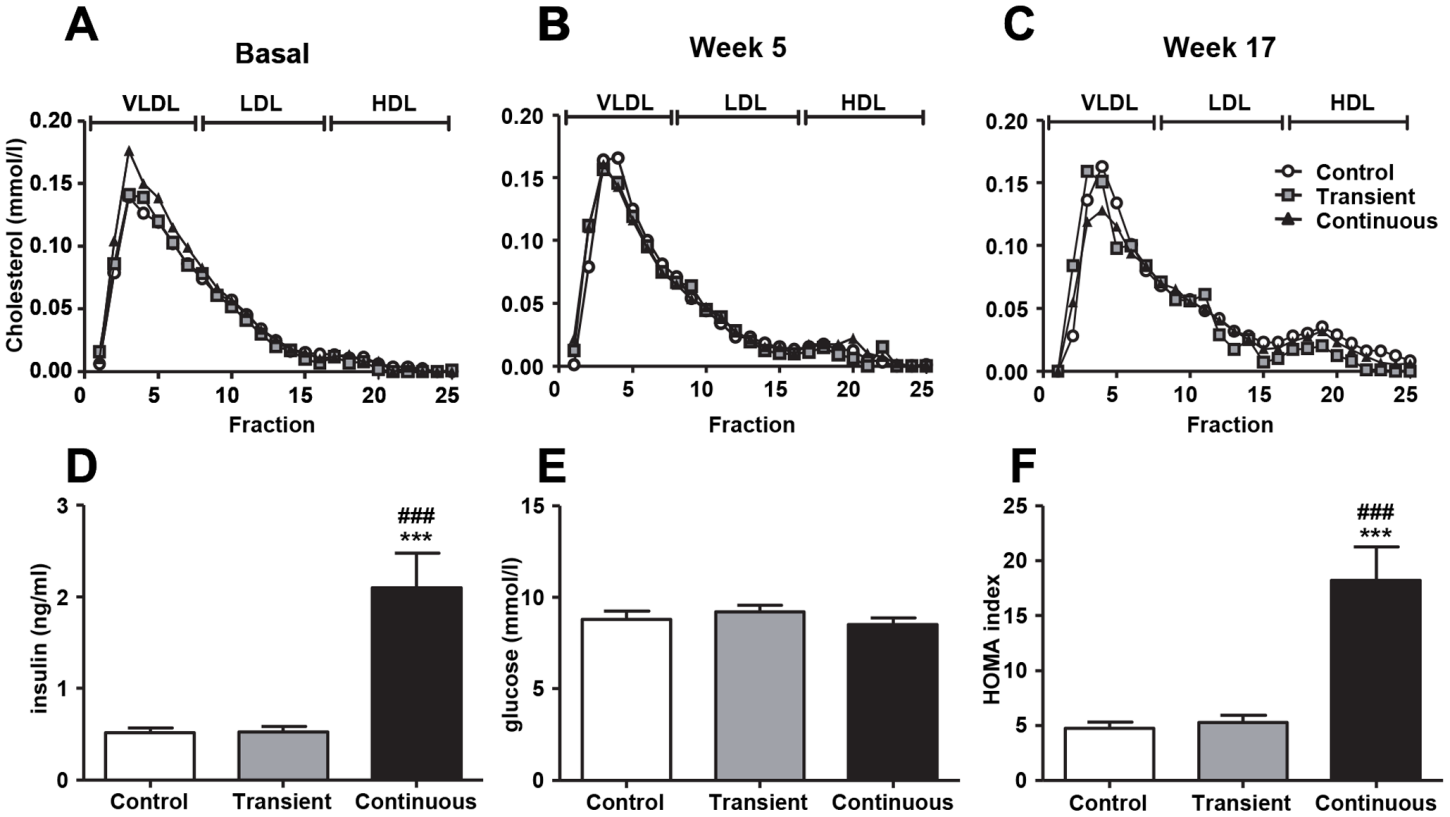
Effect of transient and continuous CORT treatment on cholesterol distribution over lipoproteins fractioned by FPLC at baseline (A), week 5 (B) and 17 (C) (Control group: white circles, transient group: grey squares and continuous group: black triangles) and on plasma insulin (D), plasma glucose (E), HOMA-IR (F), (Control group: white bars, transient group: grey bars and continuous group: black bars) on week 17. Data are means ± SEM (n = 17–21), Anova with Tukey’s post-hoc test, **^, ###^ *P*<0.001, *versus control group and ^#^versus transient group.

Although both transient and continuous administration of CORT increased food intake to a certain extent, CORT treatment did not increase plasma levels of total cholesterol, triglycerides or phospholipids during the experimental period of 17 weeks (Table 1). Moreover, there were no differences between groups in the distribution of cholesterol over lipoproteins (Fig. 3A–C). Plasma levels of insulin and HOMA-IR, but not plasma glucose levels, were increased (Fig. 3 D–F) in the continuous group at week 17, reflecting GC-induced insulin resistance.

**Table 1:** Effect of CORT treatment on fasting plasma lipids.

	Total cholesterol (mmol/)			Triglycerides (mmol/l)			Phospholipids (mmol/l)		
Week	Control	Transient	Continuous	Control	Transient	Continuous	Control	Transient	Continuous
**0**	10.0±1.5	10.2±2.3	10.2±2.1	3.9±1.1	4.3±1.1	4.1±1.4	3.6±0.4	3.7±0.6	3.5±0.6
**5**	13.2±1.8	14.0±5.2	14.8±4.2	4.6±1.6	4.8±2.3	4.5±1.6	4.5±0.6	4.5±1.1	4.6±1.0
**8**	13.4±2.0	13.7±3.9	12.4±4.3	3.8±1.5	4.0±1.7	4.2±0.9	4.5±0.5	4.6±0.8	4.2±0.9
**17**	12.4±2.8	10.2±3.2	10.0±3.0	3.2±1.4	3.3±1.0	2.9±0.5	3.8±0.9	3.4±0.9	3.4±0.7

Effect of transient and continuous CORT treatment on fasting plasma total cholesterol, triglycerides and phospholipids. Data are means ± SEM (n = 17–21), Anova with Tukey’s post-hoc test.

### Transient and Continuous CORT Treatment Decrease Atherosclerosis Lesion Area to a Similar Extent

**Figure 4:**
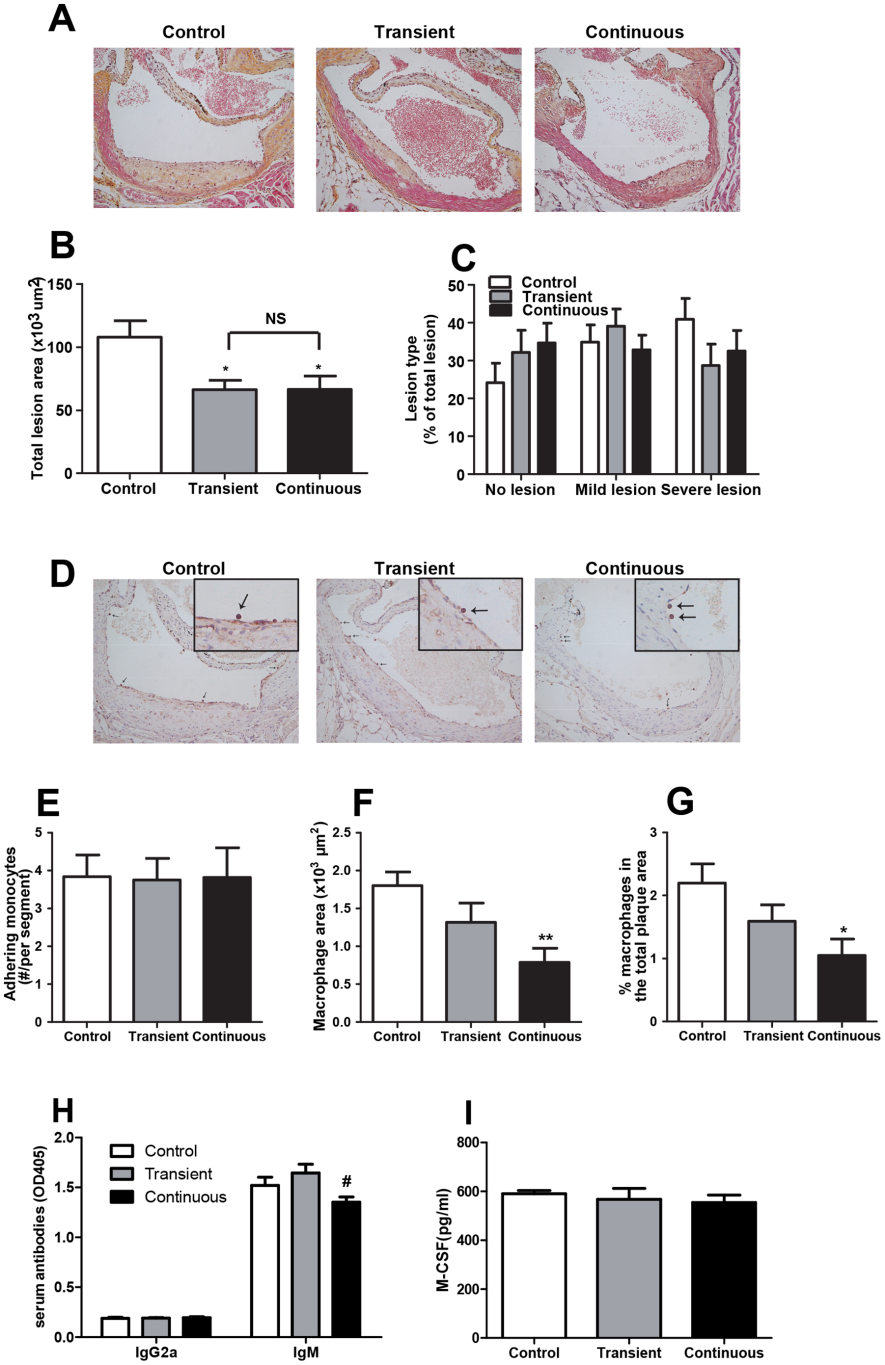
Effect of transient and continuous CORT treatment on atherosclerosis development: representative HPS-stained pictures of lesions (A), lesion area (B), lesion type as % of the total lesion (C), representative pictures of monocyte/macrophage staining (adhering monocytes shown by arrows) (D), adhering monocytes per segment (E), macrophage content of the plaque (F) and macrophages as % of the lesion area (G). Serum anti-ox-LDL specific antibodies (H) and serum M-CSF (I) were measured on week 17. (Control group: white bars, transient group: grey bars and continuous group: black bars). Data are means ± SEM (n = 17–21), Anova with Tukey’s post-hoc test, ^*#^ *P*<0.05, **P<0.01, *versus control group and ^#^versus transient group.

Remarkably, CORT treatment decreased total atherosclerotic lesion area equivalently in both transiently (−39%) and continuously (−39%) treated groups (Fig. 4A and B). Moreover, both transient and continuous groups showed similar trends towards a less severe lesion phenotype as compared to the control group (Fig. 4C), suggesting that CORT treatment reduces atherosclerosis development in a long-lasting manner. CORT treatment, neither transiently nor continuously, affected the number of monocytes adhering to endothelium wall (Fig. 4D and E), yet continuous administration of CORT did reduce the macrophage content (−56%) of the plaque (Fig. 4D and F) as well as the macrophage content as percentage of the total plaque area (−52%) (Fig. 4G). A reduction of the macrophage content of the plaque (−27%, *P* = 0.125) and percentage macrophages in total plaque area (−27%, *P* = 0.145) was observed in the transient group and but these reductions failed to reach statistical significance (Fig. 4F and G). Since uptake of oxidized LDL (ox-LDL) by macrophages to become foam cell plays an important role in the development and progression of atherosclerosis, we measured specific antibodies against ox-LDL in the circulation. As compared to control group, CORT treatment, neither transiently nor continuously, influenced the ox-LDL specific IgG2a and IgM level, indicating that CORT probably does not affect the ox-LDL level in circulation (Fig. 4H). Because macrophage proliferation and differentiation were shown to be linked to the atherosclerotic process [1], an essential factor regulating macrophage growth, macrophage colony stimulation factor (M-CSF) [30] was measured at the end of this experiment. However, no differences in the serum levels of M-CSF were detected among groups (Fig. 4I).

## Discussion

This study demonstrates for the first time that CORT treatment resulting in increased plasma CORT concentrations decreases atherosclerotic plaque formation in mice with human-like lipoprotein metabolism, without affecting either plasma lipid levels or lipoprotein profiles. Interestingly, inhibition of atherogenesis was found both in transiently and continuously exposed animals, whereas continuous treatment with CORT also decreased macrophage content of the plaque despite the normalization of the body weight. In addition, CORT treatment resulted in long-lasting changes in body fat content, which were still present even 12 weeks after abrogation of CORT. This indicates that increased CORT exposure *per se* has beneficial, long-lasting effects on atherosclerosis, but negatively affects body fat distribution and insulin sensitivity, by promoting fat accumulation in the long-term.

The concentration of glucocorticoids (GCs) attained in our experiments is higher than under stress state but well comparable to patients with severe Cushing’s syndrome, where cortisol secretion was 7 times higher that in healthy controls and circulation cortisol concentrations was 3–5 times higher [31].

Increased plasma CORT levels were easily induced non-invasively using CORT in the drinking water. CORT affected circadian GC rhythm by reducing the degree of variation in plasma concentrations. The human clinical equivalent of chronic hypercortisolemia, Cushing’s syndrome (CS), is characterized by a blunted or even complete loss of diurnal rhythm [3]. In our model we re-capitulate some of these key temporal aspects of CS: although a chronic high circulating cortisol levels is a key aspect of CS, the most reliable measure for diagnosis is very high late night (i.e. 22∶00–24∶00 h) plasma cortisol [3]. Our high- CORT animals parallel these aspects of the syndrome, with both high baseline levels of CORT (200 ng/ml), as well as a peak in CORT at the end of the night (rather than at the beginning). Moreover, the continuously exposed group displayed reduced adrenal sizes, in agreement with adrenal atrophy, secondary to the long-term exogenous CORT administration despite normal plasma CORT levels found at the end of the experiment. This might be explained by increased metabolism of CORT as well as by other pharmacokinetic adaptations like increased clearance and increased distribution volume as a result of increased adipose tissue.

Plasma lipid levels were not affected by transient nor continuous CORT treatment. This is interesting particularly in the case of plasma values of total cholesterol, VLDL-cholesterol and VLDL-triglycerides since these are well known risk factors for the development of atherosclerosis. In fact, cholesterol exposure generally is a good predictor of atherosclerosis development in E3L.CETP mice (Princen and Rensen, unpublished observations). Moreover, CORT exposure did not affect lipoprotein profiles. In previous studies, mice were subjected to chronic stress, which causes a complex of endocrine changes, including high CORT levels. These studies have reported inconsistent effects on plasma cholesterol levels, which were increased [15], decreased [16], or not reported [14]. These studies used ApoE-deficient mice in which the ApoE- deficiency causes severe accumulation of cholesterol in macrophages resulting in a high pro-inflammatory state, which could explain the differences observed in atherosclerosis development between our low grade inflammation model of E3L.CETP mice fed a low amount of dietary cholesterol (twice human daily intake) [32], [33]. In addition, the use of chronic stress to increase endogenous GCs in previous models will also induce other complex endocrine and metabolic changes [17] that may also affect atherosclerosis development. Humans with high GCs also have inconsistent cholesterol levels. In the literature, the prevalence of hyperlipidemia in patients with CS varies from 38% to 71% [34]. A study by Mancini et al (2004) has shown that hyperlipidemia occurs less frequently than the other metabolic complications of CS and that it was not correlated to the degree of hypercortisolism or duration of the disease [35]. However, the causative role of cortisol excess for hyperlipidemia has not been extensively described in the literature and the findings are, like the animal data, controversial. In some study populations, the prevalence of hypertriglyceridemia was even lower than in BMI-matched controls [36].

Intriguingly, CORT treatment reduced atherosclerotic lesion area, and tended to decrease lesion severity, to a similar extent in transiently *vs* continuously exposed mice. This suggests that GCs are able to induce long-term effects in the preliminary processes of atherosclerotic plaque formation. It is tempting to speculate on possible underlying mechanistic explanations for this phenomenon. One possibility is the induction by increased GC exposure of epigenetic mechanisms, like chromatin remodeling and histone modifications [37]. These alterations have been demonstrated in the context of chronic CORT [38], and may lead to long lasting suppression of or changes in macrophage function. Extensive documentation supports a crucial role for macrophages in the initiation of atherosclerosis by entering the vessel wall, taking up oxidized LDL (ox-LDL) [1], [2] and transforming into foam cells that produce a variety of cytokines further driving the process of the plaque formation, as well as macrophage proliferation and differentiation [39], [40]. In the present study, transient and continuous CORT treatment did not affect the plasma ox-LDL level, albeit that continuous CORT treatment significantly reduced the macrophage content of the plaque, and a similar trend was also observed in the transiently exposed group. GCs were shown to decrease the development of atherosclerosis by reducing the monocyte recruitment [41]–[43], macrophage foam cell formation [44], macrophage growth, as well as macrophage inflammatory action to produce pro-inflammatory cytokines [45]. In this study, no differences were observed in the number of monocytes adhering to endothelium between the groups. It is plausible that GCs excess inhibits macrophage growth instead of monocyte recruitment as GCs inhibit macrophage colony stimulating factor (M-CSF)-induced macrophage differentiation *in vitro* [45]. Although, we did not detect any differences in serum M-CSF level between groups, it should be realized that M-CSF is a general marker for macrophage growth, and the systemic concentration in plasma may not reflect the local macrophage growth in the vessel. Additionally, GCs can also inhibit macrophage growth by suppressing the granulocyte/macrophage colony-stimulating factor (GM-CSF) production in isolated macrophages [46], confirming that GCs might target the macrophage, the major cellular CORT target, thereby attenuating macrophage proliferation and differentiation, and thus inhibiting atherosclerotic plaque formation.

In humans, increased GC exposure, like in patients with CS, is associated with the metabolic syndrome and cardiovascular disease, even after long-term successful correction of GC excess [6], [47], [48]. Carotid intima media thickness (IMT) is increased and vessel wall plaques are more common in patients with CS [36], [49], [50]. Indeed, CS patients have abnormal fat distribution and suffer from disturbed coagulation and osteoporosis. Although, it is documented that bone mineral density fully recovers after normalization of cortisol levels, other features, like the adverse metabolic profile and the hypercoagulable state, do not completely resolve [51], [52]. The causal relation, however, between the episode of cortisol overexposure and long-term changes in the development of cardiovascular diseases is not established and is difficult to assess in humans because of the rarity and heterogeneity of CS. In agreement, in our mouse model CORT also stimulated the development of other components of the metabolic syndrome. We observed that CORT increased food intake, body weight, insulin concentrations, and altered body composition. High CORT levels are known to stimulate voluntary food intake dose-dependently [53]–[55]. GCs are also known to induce insulin resistance [48]. Moreover, insulin and CORT synergistically promote redistribution of energy storage in favor of increased fat tissue [56]. The changes in body weight were accompanied by a significant increase in both gonadal and subcutaneous fat mass in continuous group and, remarkably after 12 weeks of wash out, in transiently exposed group as well. Chronically administered GC facilitates an increase of fat mass in mice [25], [57]. In humans, increased exposure to cortisol (being either endogenous CS or exogenous corticosteroids) induces increased total body fat [58], and is specifically characterized by a redistribution of adipose tissue from peripheral to central sites of the body, mainly in the truncal region and visceral depots [59]. This is accompanied by a greater than two-fold increased risk in insulin resistance/diabetes, hypertension, and hyperlipidemia [39]. In the human equivalent of severe chronic stress, CS, the prevalence of the metabolic syndrome is increased [47], [48], and intriguingly, after remission, these patients still have increased waist circumference [36], [60] and higher visceral fat mass without an effect on the body mass index [61], and their cardiovascular risk remains increased.

Adipose tissue macrophages are the primary source of the proinflammatory cytokines, and the macrophage content of adipose tissue has been shown to correlate positively with adiposity [62]. In the present study, although excess GC exposure increased fat mass and induced obesity, which was accompanied with an increase in macrophage content, inflammation in the adipose tissue was not elevated. Moreover, after abrogation of CORT treatment, the macrophage content in the adipose tissue was normalized and the reduction of inflammation was persisted in the long-term despite of the presence of a persistent increase in fat mass. CORT treatment induces prolonged, complex changes in the adipose tissue that reflect both the adverse metabolic effects of glucocorticoids by increasing the adiposity, and the anti-inflammatory capacity by reducing the expression of the proinflammatory molecules in the macrophages. Therefore, we cannot exclude that the protective effects of CORT treatment on atherosclerosis development can, at least partly, be explained by decreased release of cytokines from adipose tissue. It is well possible that at high dose, the anti-inflammatory effects of GCs attenuate potentially adverse metabolic influences. In clinical practice, this means that GC schemes as used for anti-inflammatory indications might benefit from adjustments towards a higher dose for a shorter period of time (or even as a few ‘high dose ‘pulses’ as is used in clinical practice with methyprednisolone instead of lower dosages for a prolonged period of time.

In conclusion, increased CORT exposure in mice with a human like lipoprotein metabolism has beneficial, long-lasting effects on atherosclerosis, despite negatively affecting body fat distribution and insulin sensitivity by promoting fat accumulation in the long term. This indicates that the increased atherosclerosis observed in the human in states of GC excess may not be related to cortisol *per se*, but may be the result of complex effects of cortisol on the endothelium and/or coagulation. The effects of GC excess, therefore, are multiple, and dependent on many factors, but above all, may irreversibly affect many pathophysiological processes, thereby influencing long-term cardiovascular risk.

## Supporting Information

Table S1 **Primer sequences used for RT-qPCR.**
(DOC)
