# Supplementary material for: Both Transient and Continuous Corticosterone Excess Inhibit Atherosclerotic Plaque Formation in APOE*3-Leiden.CETP Mice
Source: PLoS One. 2013 May 22;8(5):e63882. doi: 10.1371/journal.pone.0063882 (PMC3661690; doi:10.1371/journal.pone.0063882)
Supplement: Table S1 — Primer sequences used for RT-qPCR. (DOC) [file pone.0063882.s001.doc]

Supplement to

**Both transient and continuous corticosterone excess inhibit atherosclerotic plaque formation in APOE*3-Leiden.CETP mice**

Hanna E. Auvinen^a,d*^, Yanan Wang^a,d*^, Hans Princen^b^, Johannes A. Romijn^a,c^, Louis M. Havekes^a,b,d^, Johannes W.A. Smit^a^, Onno C. Meijer^a,d^, Nienke R. Biermasz^a^, Patrick C.N. Rensen^a,d^ and Alberto M. Pereira^a^

^a^Department of Endocrinology and Metabolic Diseases, Leiden University Medical Center, P.O. Box 9600, 2300 RC Leiden, The Netherlands.

^b^TNO Metabolic Health Research, Gaubius Laboratory, P.O. Box 2215, 2333 CK Leiden, The Netherlands

^c^Department of Medicine, Academic Medical Center, P.O. Box 22660, 1100 DD Amsterdam, The Netherlands

^d^Einthoven Laboratory for Experimental Vascular Medicine, Leiden University Medical Center, P.O. Box 9600, 2300 RC Leiden, The Netherlands.

*Both authors have contributed equally

**Abbreviated title:** High CORT and Atherosclerosis in E3L.CETP Mice

**Supplemental Table 1. Primer sequences used for RT-qPCR**

| **Gene** | **Forward primer** | **Reverse Primer** |
| --- | --- | --- |
| *β2m* | TGACCGGCTTGTATGCTATC | CAGTGTGAGCCAGGATATAG |
| *Cd68* | ATCCCCACCTGTCTCTCTCA | TTGCATTTCCACAGCAGAAG |
| *F4/80* | CTTTGGCTATGGGCTTCCAGTC | GCAAGGAGGACAGAGTTTATCGTG |
| *Hprt* | TTGCTCGAGATGTCATGAAGGA | AGCAGGTCAGCAAAGAACTTATAG |
| *Il-6* | TGTGCAATGGCAATTCTGAT | CTCTGAAGGACTCTGGCTTTG |
| *Tnfα* | AGCCCACGTCGTAGCAAACCAC | TCGGGGCAGCCTTGTCCCTT |

*β2m,* β2-microglobulin; *Hprt,* hypoxanthine ribosyltransferase; *IL-6*, Interleukin-6; *Tnfα,* Tumor necrosis factor α.
